# Diverging drivers of fungal diversity: seasonal effects shape aboveground communities, while geographical patterns govern belowground communities in rubber tree ecosystems

**DOI:** 10.1128/spectrum.00458-25

**Published:** 2025-05-23

**Authors:** Gareth Trubl

**Affiliations:** 1 Physical and Life Sciences Directorate, Lawrence Livermore National Laboratory, Livermore, California, USA; Ruhr-Universitat Bochum, Bochum, Germany

**Keywords:** fungal diversity, spatiotemporal drivers, phyllosphere, leaf endosphere, soil, rhizosphere, root endosphere

## Abstract

Understanding the spatiotemporal dynamics of microbial communities is essential for predicting their ecological roles and interactions with host plants. In a recent study, Wei and colleagues (Microbiol Spectr 13:e02097-24, 2024) investigated fungal diversity across multiple plant and soil compartments in rubber trees over two seasons and two geographically distinct regions in China. Their findings revealed that alpha diversity was primarily influenced by seasonal changes and physicochemical factors, while beta diversity exhibited a strong geographical pattern, shaped by leaf phosphorus and soil available potassium. These results highlight the role of environmental drivers in shaping within-community diversity, while other factors contribute to the differences between fungal communities across the soil–plant continuum. By distinguishing the effects of temporal and spatial factors, this study provides detailed insights into plant-associated microbiomes and emphasizes the need for further research on the functional implications of microbial diversity in the context of changing environmental and agricultural conditions.

## COMMENTARY

The relationship between environmental factors and microbial diversity, especially in soil and plant-associated microbiomes, has long been a key area of study. Fungi are vital components of these microbiomes, playing essential roles in nutrient cycling and overall ecosystem functioning (1). As one of the most abundant and diverse groups of microbes in soils, fungi decompose complex organic materials, form mycelial networks, and help maintain soil structure and water retention. Their interactions with bacteria—ranging from mutualistic to antagonistic—shape microbial community dynamics and influence biogeochemical cycles (2). Understanding the environmental drivers, such as seasonal and spatial factors, that influence fungal community structure is important for enhancing our understanding of microbial diversity and its broader implications for ecosystem health. The study by Wei and colleagues (3) provides new insights into the environmental factors that shape fungal communities by examining the rubber tree soil–plant continuum (sampling the phyllosphere, leaf endosphere, soil, rhizosphere, rhizoplane, and root endosphere). Their findings highlight the influence of both seasonal and spatial factors on microbial diversity.

A central finding of this study is the contrast between fungal alpha and beta diversity in the rubber tree soil–plant continuum. Alpha diversity was found to be highly responsive to seasonal changes, particularly temperature and precipitation. This is consistent with a prior study at Hainan Island in China showing that seasonal variations in temperature and moisture availability drive fluctuations in microbial diversity across different environments (4). The authors show that these environmental drivers are important in aboveground compartments, such as the leaf endosphere and phyllosphere, where fungal communities may be more directly influenced by atmospheric conditions. In contrast, the group’s previous work (5), which focused solely on the soil microbiome across Hainan Island, found that spatial factors outweighed seasonal effects. This suggests that soil microbial communities may be more resistant to seasonal fluctuations due to the buffering effects of edaphic conditions, whereas aboveground fungal communities are more sensitive to changes like temperature and precipitation. Further, while richness increased in some compartments during the dry season, Shannon’s diversity and evenness remained unchanged. This suggests that the new fungal taxa likely filled available niche space without significantly altering the relative abundances of existing taxa. The taxa may have maintained their proportions, along with new taxa, adding to overall richness but not shifting the overall community structure. This suggests seasonal shifts may expand fungal community membership without altering community structure.

While the within-community diversity was influenced by seasonal changes, the beta diversity of fungal communities was primarily influenced by geographical location. This suggests that historical factors, soil properties, and site-specific environmental conditions play a dominant role in structuring fungal community composition across large spatial scales. The study identified phosphorus in leaves and available potassium in soil as key contributors to spatial variation in beta diversity. In their earlier work on Hainan Island soil microbial communities, the group found that fungal communities were more stochastic in the rainy season, with soil properties like pH and potassium shaping community assembly (6). Building on these findings, the current study reinforces the role of deterministic processes in shaping fungal communities by identifying phosphorus and potassium as key drivers of fungal beta diversity.

This study supports broader ecological theories suggesting that microbial diversity follows predictable patterns along environmental gradients (7). In terms of beta diversity, the stronger influence of geographical location aligns with biogeographical studies suggesting that microbial community composition is shaped by local soil conditions, plant host factors, and historical influences. These findings support the idea that microbial diversity is controlled by both climatic and historical contexts, with microbial communities adapting to specific gradients of temperature or soil pH (8). The authors propose that leaf phosphorus and soil potassium are key factors shaping fungal community dissimilarity across sites. These nutrients, which affect plant metabolism and defense mechanisms, may influence fungal community structure through host-mediated selection pressures. This highlights the complex interaction between plant traits, soil nutrients, and microbial community dynamics, underscoring the importance of considering both environmental and historical factors in understanding microbial diversity.

Microbiomes are inherently complex, and understanding their temporal trends remains challenging due to the limitations of our sampling capabilities, which capture only a snapshot in time. Additionally, determining the appropriate spatial scale for measurements is difficult, as we are constrained by the size of samples and the extent to which we can characterize them. Machine learning offers a powerful solution to these limitations, enabling the integration of diverse microbiome data and the identification of underlying trends and key features that might otherwise be overlooked (9). In this study, the use of machine learning, specifically random forest analysis, to identify the environmental drivers of soil fungal alpha diversity is an excellent way to use machine learning for microbiome analyses. Through random forest analysis, the authors successfully pinpoint critical variables that influence microbial communities, revealing machine learning’s ability to uncover complex, nonlinear relationships between biological and environmental data. This approach allows for a deeper understanding of how factors such as soil properties, leaf characteristics, and other environmental features interact to shape fungal communities across both aboveground and belowground compartments. Additionally, the study’s use of variation partitioning analysis to assess the relative contributions of seasonal changes, environmental conditions, and geographical location further illuminates the intricate dynamics of microbial community assembly.

The study by Wei and colleagues (3) offers valuable insights into the environmental drivers of fungal diversity across plant and soil compartments, shedding light on the temporal and spatial factors that shape microbial communities in the rubber tree soil–plant continuum. The distinction between alpha and beta diversity patterns highlights the importance of considering both seasonal influences on within-community diversity and geographical factors in shaping community composition. The role of phosphorus in leaves and potassium in soil as key contributors to spatial variation in beta diversity highlights the complex interactions between plant traits, soil nutrients, and fungal communities. Additionally, the application of machine learning techniques to identify critical environmental drivers and uncover complex relationships in microbial data enhances the integration of current findings with previous research, providing a more comprehensive understanding of fungal community dynamics. This approach also sheds light on the broader implications of microbial diversity for ecosystem stability and sustainability, especially in the face of changing environmental conditions and agricultural practices.
